# Caregiver and provider perspectives on developmental services for children with sickle cell disease: a mixed methods analysis

**DOI:** 10.3389/fped.2025.1530457

**Published:** 2025-03-21

**Authors:** Mollie Tamboli, Erin MacArthur, Natalie Collins, Eunyoung Kang, Maria Fernandez, Jerlym S. Porter, Heather M. Conklin, Allison A. King, Jane S. Hankins, Andrew M. Heitzer

**Affiliations:** ^1^ Departments of Psychology and Biobehavioral Sciences, St. Jude Children’s Research Hospital, Memphis, TN, United States; ^2^ Institute for Implementation Science, University Texas Health Science Center at Houston, Houston, TX, United States; ^3^ Departments of Occupational Therapy, Neurology, and Pediatrics, Washington University in St. Louis, St. Louis, MO, United States; ^4^ Departments of Global Pediatrics, St. Jude Children’s Research Hospital, Memphis, TN, United States; ^5^ Departments of Hematology, St. Jude Children’s Research Hospital, Memphis, TN, United States

**Keywords:** sickle cell, mixed-methods, intervention, developmental, caregivers

## Abstract

**Introduction:**

Sickle cell disease (SCD) is a monogenic blood disorder characterized by neurodevelopmental delays. Most children with SCD do not receive developmental services due in part to disparities in care access. To inform the design of a developmental intervention for children with SCD, we evaluated factors that influence access to developmental services.

**Methods:**

Interview data were collected from educational and medical providers (*n* = 15) and caregivers (*n* = 15) of children aged 4–6 years with SCD at a single center and the surrounding area. Caregivers completed questionnaires about their child's background/medical history, caregiver depression (PROMIS SF v1.0-8a), and caregiver knowledge of early development (Knowledge of Infant Development Inventory). A convergent design was used to integrate the qualitative and quantitative data.

**Results:**

We identified three themes as factors that influence caregivers' access to developmental services: quality of medical and educational experiences, caregiver knowledge and beliefs about SCD and development, and caregiver preferences for developmental services. Most caregivers denied barriers to obtaining developmental services for their child, whereas providers acknowledged numerous barriers for families. Caregivers and providers shared that a positive caregiver-provider relationship facilitates access. Caregivers reported that there was limited attention to SCD within the hospital system and broader society. Caregivers displayed limited knowledge of early development, and providers identified these knowledge gaps as a barrier to utilizing developmental services. Caregivers expressed a strong interest in SCD education and community building.

**Conclusions:**

Our mixed method analysis identified barriers and facilitators to developmental services for children with SCD.

## 1 Introduction

Sickle cell disease (SCD) is a monogenic blood disorder that impacts nearly all systems of the body (1). Approximately 100,000 individuals are estimated to have SCD in the United States, the majority of whom are African American (2). The brain is particularly vulnerable to the effects of SCD due to poor oxygen delivery, resulting in cerebrovascular insults (3–5) and accelerated white matter loss (6). Early and progressive neurocognitive deficits are often seen in patients diagnosed with SCD, negatively impacting academic performance (7, 8).

Individuals living with SCD have less access to comprehensive care compared to individuals with other chronic diseases due to a lack of SCD-trained providers and health insurance barriers (9). These disparities translate to low rates of preventive care, such as immunizations (10) and transcranial doppler screening for stroke prevention (11). Disparities in healthcare access extend to developmental services that may remediate developmental delays in young children with SCD, and better prepare them for academic success. The academic consequences are dire, as children with SCD fall below normative expectations on achievement assessments and have a high rate of grade retention (12). Developmental services include speech, occupational, physical and other early intervention therapies, as well as classroom-based interventions. Because neurocognitive deficits become more apparent with age (13, 14) and early intervention tends to be more effective (15, 16), developmental services for SCD should use a preventive approach to alter the developmental trajectory rather than a corrective approach after functional impairment occurs (17, 18). However, despite the benefits of early interventions, most children with SCD do not receive developmental services to address delays (19).

Many families with a child with SCD experience a double burden as members of a historically marginalized group coping with chronic illness (20). Although some facilitators and barriers are known to impact medical care for SCD families, no studies have provided a comprehensive view from both caregivers and providers of determinants affecting utilization of developmental services. Reported facilitators to medical care include convenience of combined appointments (i.e., having subspecialists knowledgeable about SCD present together) and the ability to communicate with knowledgeable staff about SCD concerns (21). Reported barriers to medical care include perceived discrimination, the distance from and transportation to SCD centers, financial strain, missing work, children missing school, and lack of provider knowledge and comfort (21, 22). Previous studies offer insight into barriers into medical care, but do not provide insight into barriers to developmental care, which differs from medical care in a variety of ways, not limited to treatment location, referral process, and provider training. There is a significant need to implement developmental interventions for children with SCD that are both feasible and acceptable as prior intervention trials have struggled with engagement and adherence (23, 24). A better understanding of caregivers' access to and willingness to obtain developmental services could help providers tailor services to the SCD population.

This investigation uses the Health Equity Implementation Framework (25) (HEIF) to identify determinants of access to developmental services specific to the SCD community. Unlike previous studies on facilitators and barriers that impact care (21, 22), this study incorporates both caregiver and provider perspectives. We used a convergent mixed methods design (26) to better understand what factors impact access to and preferences for developmental services. The current study has three aims: (1) identify barriers and facilitators to developmental treatment faced by caregivers of children ages 4–6 with SCD; (2) understand which factors inform decisions about medical, developmental, and educational interventions for caregivers of young children with SCD; and (3) identify caregiver preferences that will increase developmental treatment utilization for young children with SCD.

## 2 Materials and methods

### 2.1 Participants and procedures

Medical and educational providers and caregivers of children with SCD ages 4–6 were recruited from the Memphis area. Snowball sampling was used to find providers familiar with the unique developmental needs of young children with SCD, starting with providers at St. Jude Children's Research Hospital and a local early childhood education provider. Providers at these organizations recommended colleagues with experience providing or connecting families to developmental services, and/or experience working with families of young children with SCD. Caregivers were recruited through the Sickle Cell Clinical Research and Intervention Program (SCCRIP), a longitudinal cohort study of patients with an SCD diagnosis (27). Caregivers were eligible if they spoke English and had a child enrolled in the cohort study between the ages of 4 and 6 years with any SCD genotype.

This was a descriptive cross-sectional study. We used a convergent mixed methods design, involving parallel collection of both quantitative and qualitative data. This approach was used as both quantitative and qualitative domains of interest were identified *a priori*, consistent with the HEIF framework (25). All HEIF domains were examined qualitatively and quantitatively either through frequency counts or formal questionnaires. Certain themes arose from interviews that were not captured quantitatively, and interpretation was primarily based on qualitative data collected. Interviews with providers were conducted over a virtual video platform or over the phone and were audio recorded. All caregiver interviews were conducted over the phone and audio recorded. Audio recording of interviews was granted by participants during the informed consent process. Interviews were transcribed verbatim using a third-party service and checked for accuracy by two study team members. Caregivers also completed four questionnaires: two descriptive questionnaires about their child's background and medical history, a questionnaire about caregiver depression, and a questionnaire testing the caregiver's knowledge of early development. The procedures of this study were approved by the Institutional Review Board at St. Jude Children's Research Hospital.

### 2.2 Qualitative interviews

Two semi-structured interview guides, one for caregivers and one for providers, were developed based on the HEIF used by Woodward et al. (25) to study barriers and facilitators to treatment for the hepatitis C virus. The HEIF integrates healthcare disparities and implementation science concepts into a framework that considers barriers and facilitators to intervention implementation at the following levels: recipients (patients and providers), clinical encounters, innovation/characteristics of treatment, and the context of the health care system. This framework allows for the identification of disparities in access to care and racially disparate decisions by providers (28). Caregivers were asked about potential barriers and facilitators to developmental services for their child with SCD. Caregivers with children who had not received developmental services were asked what they would expect from a developmental service based on their experience with their child's medical treatment or education. Providers were asked the same questions and answered based on their knowledge of developmental services and their interactions with caregivers. Additional questions for caregivers were included to learn about their knowledge of SCD and development and their preferences for a proposed intervention (see Supplementary File 1 and Supplementary File 2 for interview guides).

### 2.3 Quantitative measures

#### 2.3.1 Depression

The Patient Reported Outcomes Measurement Information System (PROMIS) is a collection of item banks made by the National Institute of Health and includes a depression questionnaire about negative mood, decrease in positive affect, information processing deficits, negative views of the self, and negative social cognition. The PROMIS SF v1.0 - Depression 8a, an 8-item depression short form, is highly correlated to the full depression bank (*r* = 0.96) (29). Items have five response options (Never, Rarely, Sometimes, Often, and Always), and response pattern scoring is used to calculate a raw score that is transformed into a *T*-score with a general population mean of 50 and standard deviation (SD) of 10. A *T*-score above 60 indicates moderate depression, and a *T*-score above 70 indicates severe depression (30). The general population used to norm the *T*-scores (*n* = 11,796) is similar in gender, age, race/ethnicity, and education to the 2010 U.S. census (31).

#### 2.3.2 Knowledge of development

The Knowledge of Infant Development Inventory (KIDI) is a 58-item questionnaire that assesses parent knowledge of infant norms and milestones, principles of development, parenting strategies, and health and safety (32). Item responses include Agree, Disagree, and Not sure for 39 questions, and Agree, Younger, Older, or Not sure for 19 questions that make a declarative statement like “Babies say their first real word at 6 months.” Two response scores were calculated (Attempted and Accuracy). The KIDI's internal consistency across a diverse sample from 10 studies (*N* = 820) is acceptable (*α* = 0.88) (32). A study by Hamilton and Orme (33) found that the KIDI had high convergent validity with the Knowledge of Child Development Inventory (*r* = 0.68) and the Parent Opinion Question (*r* = 0.51).

#### 2.3.3 Demographic and medical information

A demographics questionnaire and a medical history questionnaire were used to gather background information about the sampled caregivers. The demographics questionnaire asked about caregiver characteristics including education, marital status, income, health care coverage, race, and ethnicity. The medical history questionnaire asked about the child's SCD-related complications and treatment, prenatal and birth history, and developmental and educational history.

### 2.4 Qualitative analysis

A qualitative content analysis was performed on the interview data to identify descriptive themes. After the data were transcribed and assessed for quality, each transcript was coded by two independent coders (MT and EM) using Delve Tool software (34). Two coders used a deductive approach for questions derived from the HEIF (25) and questions about caregiver preferences on the proposed interventions. First, a codebook was created that aligned with the barrier and facilitator domains of the HEIF and questions specific to medical experiences and preferences. In addition to the *a priori* codes, other codes for recurring topics discussed throughout the interviews were inductively derived, including “Caregiver decision making” and “Beliefs about learning and academics.” The two coders met during and after their independent coding processes to discuss their findings and resolve coding discrepancies.

After the codes for each transcript were agreed upon by the two coders, the data were visually reorganized by code in a categorization matrix (35). The categorization matrix is an arrangement of summarized participant responses with a row for each participant and a column for each code. The matrix enabled condensing of the data (i.e., whether a barrier was or was not endorsed, and whether a facilitator was identified). The data were further abstracted from the matrix as the coders analyzed the code columns to write an interpretive summary of responses. Responses were also analyzed by row to identify patterns within individual responses. Analysis of the interpretive summaries allowed for the re-contextualizing of data into descriptive themes that described the experiences, beliefs, and preferences of providers and caregivers (36). To further analyze within each theme for subthemes, responses were transformed into frequencies of endorsed barriers and facilitators as detailed below.

### 2.5 Quantitative data analysis

Responses to barrier questions derived from the HEIF were quantified by dichotomizing responses that did or did not endorse a specific barrier and analyzed for frequencies. Responses to the inductively-derived codes of “Medical knowledge of SCD,” “Knowledge of SCD and development,” “Sources of knowledge,” “Perceptions of society,” and “Decision making” were quantified for the frequency of topics discussed within each code. For the PROMIS, descriptive statistics were calculated for caregiver responses. For the KIDI, an accuracy score was calculated from the ratio of the total correct of the total attempted items per participant. Mean and SD were used to analyze the accuracy and over/underestimate scores from the KIDI. For the medical and demographic questionnaires, frequencies were used to describe the categorical data and mean and SD were used to analyze the continuous data.

### 2.6 Data integration

A convergent design was used in this mixed methods analysis (26), beginning with the independent analysis of qualitative and quantitative data, followed by the integration of the two data sets to identify ways in which the two methods of caregiver reporting confirm, contradict, or expand on caregiver experiences. The transformation of interview responses into frequencies informed the construction of descriptive themes. A basic joint display (37) report transformed interview data, interview quotes, and survey data, while also showing the results in the context of the HEIF domains.

## 3 Results

Fifteen providers with a range of 5–29 years of experience in the medical, education, and/or advocacy sectors participated in interviews. Roles of the providers included hospital-school coordinators who advocate for medical and educational needs (*n* = 4), hospital teachers (*n* = 2), school leadership from the hospital and the community (*n* = 4), early childhood health and disability specialists (*n* = 2), an adult SCD advocate, a hematologist, and a hematology administrator. Seventeen caregivers were enrolled and fifteen caregivers completed surveys and interviews (2 caregivers were lost to follow up). Caregivers ranged in age from 23 to 40 years (Mean = 29.4), were primarily female (94%), and all identified as Black or African American. Most caregivers had children who attended preschool (80%) and/or received a developmental service (60%). Characteristics of the caregivers and their children can be found in Table 1.

**Table 1:** Caregiver and child characteristics.

Caregiver	*n* (%)
Mean age in years [range]	29.4 [23–40]
Gender	
Female	14 (94)
Male	1 (6)
Race	
Black or African American	15 (100)
Ethnicity	
Not Hispanic or Latino	12 (87)
Unknown/no answer	3 (13)
Maternal highest education	
High school education/General Education Diploma or less	6 (40)
Some college or associates	6 (40)
College	1 (6.7)
Graduate degree	2 (13.3)
Primary Insurance: state or federal Insurance	12 (80)
Caregiver annual income	
Less than $25,000 USD	6 (40)
$25,000–$50,000	3 (20)
$51,000–$75,000	2 (13.3)
$76,000–$100,000	0 (0)
$101,000–$150,000	2 (13.3)
Unknown/no answer	2 (13.3)
Depression	
Mean PROMIS T-Score [range]	46.1 [38.2–64.2]
Knowledge of development	
Average % correct KIDI responses [range]	61% [50%–71%]
Child	*n* (%)
Mean age in years [range]	4.8 [4–5]
Gender	
Female	9 (60)
Male	6 (40)
Race	
Black or African American	15 (100)
Ethnicity	
Not Hispanic or Latino	12 (80.0)
Hispanic or Latino	1 (6.7)
Missing	2 (13.3)
SCD genotype	
SS	8 (53.3)
SC	6 (40)
Not sure/no answer	1 (6.7)
Education and services	
Preschool/Head Start	12 (80)
Developmental services^a^	9 (60)

KIDI, Knowledge of Infant Development Inventory; PROMIS, Patient Reported Outcomes Measurement Information System; SCD, sickle cell disease.

^a^ Developmental services includes early intervention services such as occupational, speech, physical, and developmental therapy.

**Figure 1:**
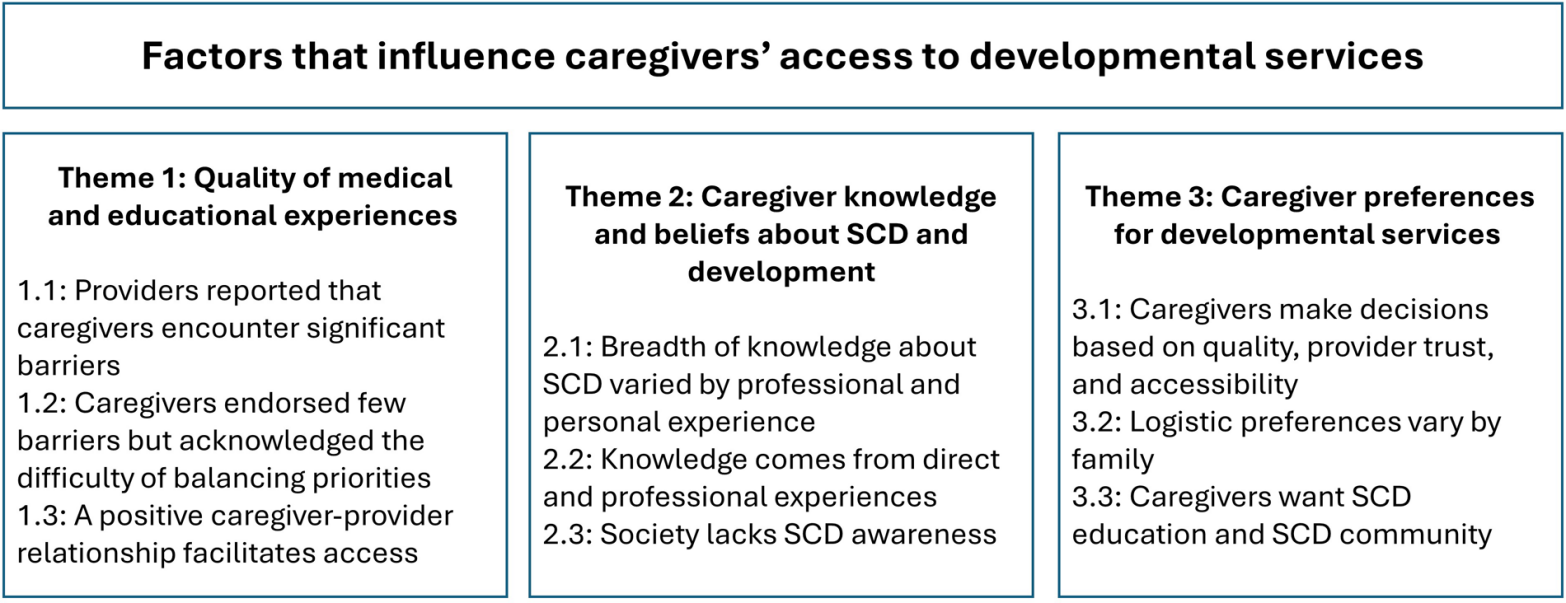
Results of the thematic analysis. The themes represent factors that influence caregivers’ access to developmental services.

The study team identified three themes as factors that influence caregivers' access to developmental services: quality of medical and educational experiences, caregiver knowledge and beliefs about SCD and development, and caregiver preferences for developmental services (Figure 1).

### 3.1 Theme 1: quality of medical and educational experiences

#### 3.1.1 Providers reported that caregivers encounter significant barriers

When asked about specific barriers to developmental care, most providers endorsed at least some of the barriers within each HEIF domain (Table 2). The barriers most frequently endorsed were the patient's housing or living situation (*n* = 14), caregivers' unawareness that treatment exists (*n* = 13), lack of media attention around treatment (*n* = 12), caregiver stress or depression (*n* = 12), and lack of transportation (*n* = 12). Providers also shared experiences of caregivers not understanding a developmental diagnosis or treatment and/or not believing a treatment was necessary.

**Table 2:** Caregiver experience with barriers to developmental treatment.

HEIF domain	Selected interview and survey questions	Endorsed by providers *n* (%)	Endorsed by caregivers *n* (%)	Participant-generated barriers	Provider quotes	Caregiver quotes
Intervention (characteristics of treatment)	Caregivers were unaware that treatment exists	13 (86.7)	0 (0)	• Appointments take place during the caregiver's work hours • Caregivers unhappy that their child did not qualify for services • School documentation associated with diagnosis and treatment (i.e. Individualized learning plans) is time consuming	“Making those services available with more flexible hours… some of the families… like their work hours look different. So if we're going to try to provide a service… how can we fit that into what our families are, you know, what they can do.”	“They tried their very best, you know, like, as far as helping him and get him to the point where he needs to be. And I understood that it just, you know, it just takes time.”
	The cost of treatment is prohibitive	5 (33.3)	3 (20)			
	Caregivers struggle with wait times to get an appointment	10 (66.7)	2 (13.3)			
Clinical encounters	Problems when the patient met with a developmental provider	5 (33.3)	0 (0)	• Caregivers felt uncomfortable asking questions • Provider didn't value input from caregiver	“"I think… Listening, right? I think that it's, it's important to most people to feel seen and heard, um, and to feel like the things that matter to them matter to the people that are, are charged with taking care of them.”	“I have learned that if you’ve never experienced what I experienced or deal what I had to deal with, you really wouldn't understand… You know, being patient and, you know, trying to, you know, understand to a, uh, certain point.”
	Caregiver felt or would expect to feel misunderstood by a provider	8 (53.3)	2 (13.3)			
Patient factors	Caregiver's motivation to seek treatment acts as a barrier	7 (46.7)	1 (6.7)	• Family dynamics can impact treatment • Family's relationship to the school district may impact perception of treatment • Multiple children/ family members with different needs to care for • Inability to take time off at work for appointments	“our parents have lives to live. And so this is a chronic illness… So if you have three other children, and you’re trying and they’re all in school, and they all have different activities, and you’re trying to work. And so if someone says, come up to [Children's Hospital] twice a week, you know, for an hour at a time, uh, that's not feasible for a lot of people.”	“Sometimes it's hard to… like juggle bills with that, um, with having to travel back and forth like to [mid-south city] and stuff like that, um, for doctor's appointments and stuff like that.”
	Trust in providers (or lack of trust) acts as a barrier	8 (53.3)	2 (13.3)			
	Caregiver stress or depression acts as a barrier	12 (80)	3 (20)			
	Child has experienced SCD related pain^a^		13 (86.7)			
	Child had a SCD related hospitalization^a^		12 (80)			
	Child has other health conditions^a^		2 (13.3)			
Provider factors	Providers were too busy to provide quality care	7 (46.7)	1 (6.7)	• Lack of diversity among providers • Providers talking too quickly or with too much jargon • Providers lack of knowledge about SCD	“I have had parents to say, well, the meeting doesn't ever go well if you're not there, because I don't know what they're saying. They're talking too fast.”	“I think in the process of trying to get a person for that service, uh, in relation to my culture, me being black… probably more so wanting someone of a similar background. So that some things culturally would make more sense to that individual…”
	Providers did not answer the patient's questions	6 (40)	1 (6.7)			
Community resources	Limited providers available in the community	10 (66.7)	1 (6.7)	• Low income schools are under-performing, which impacts access to services • Caregivers with public insurance may need to go through the school system to get developmental services	"I think zip code matters, depending on where you live determines what kind of treatment you get. Maybe there's patients that don't even have a school in their town, they have to go to the next town.”	“Yeah, I, I would expect to have difficulties just because we live in a rural community and it's not as many, um, specialized physicians in the area.”
	Harder to get treatment in the community	11 (73.3)	1 (6.7)			
Perception of society	In society, there's a lack of caring about patients living with SCD	4 (26.7)	5 (33.3)	• Teachers know less about SCD than they used to • Bias against SCD in daycares, hospitals, and schools • Caregivers have the burden of educating others about SCD • SCD is not a priority in society because of racism	“Most of the people with money and resources don't look like the people with sickle cell disease. So that means that resources just, uh, like I said, I don't know if it's a – I can't say it's a lack of caring about sickle cell disease, but more about other priorities.”	“I feel like people rarely talk about that. Some people don't even know what that is. They don't know what sickle cell is.”
	There is a lack of media coverage about SCD	12 (80.0)	11 (73.3)			

In column 2, interview questions were condensed into statements. Columns three and four indicate endorsement of that statement by participant group: stakeholder or caregiver. HEIF, Health Equity Implementation Framework; SCD, sickle cell disease.

^a^ Questions from the medical history questionnaire were answered only by caregivers.

Some providers further stated that specific barriers are related to systemic issues in hospitals and schools. One such issue is miscommunication between providers and caregivers about why a child needs services or how services can be helpful. This issue arises particularly when providers speak too quickly or use jargon. One health services specialist working in early childhood education explained how she helps caregivers ask questions:

“We actually have forms that we give to parents that, that ask doctors specific questions.. we have found that some parents do not know what to ask. I’ve actually had parents – I say, ‘Well, when you get there, this is my cell number. Call me and hand the phone to the doctor.’” (Provider 16)

Another barrier expressed primarily by education providers is caregiver mistrust of the medical or educational system. Educators described conversations with caregivers who associate early intervention with the stigma around special education. One educator explained that caregivers who received special education services before schools adopted inclusion models may avoid developmental services out of fear that their child may be labeled or ostracized.

Providers also discussed health and socioeconomic inequalities faced by caregivers of children with SCD and how such inequalities impact caregiver motivation, mental health, and trust in developmental providers. One provider explained how low caregiver wellbeing impacts access to services:

“The stress, you know, you have so many stress factors, living in the high crime area, living in a food desert, not having a car, uh, living in an abusive situation, not having enough money to buy food. Yeah. I mean, of course… your hierarchy is not about services for your child; it’s about everyday existence.” (Provider 12)

#### 3.1.2 Caregivers endorsed few barriers but acknowledged the difficulty of balancing priorities

Unlike providers, most caregivers endorsed few, if any, of the HEIF barriers (Table 2). For example, most providers endorsed some or all of the barriers in the patient factor of the HEIF domain, but the only patient-factor barriers that were endorsed by more than one caregiver were distance from the hospital or clinic (*n* = 3), transportation (*n* = 3), trusting providers (*n* = 2) and experiences of stress or depression (*n* = 3).

To better contextualize caregiver responses to the patient-factor HEIF questions, we asked caregivers to complete a brief depression symptom inventory (PROMIS SF V.1- Depression 8a) Caregiver scores ranged from 38.2 to 64.2 with a mean of 46.1 (SD = 9.4), which is below the standardized *T*-score of 50 in the general population. Two caregivers (13.0%) scored in the clinical range for depressive symptoms. Another patient factor that may impact access to developmental services is the severity of the child's SCD symptoms. The majority of caregivers reported that their child had experienced SCD-related pain (*n* = 13, *M* = 86.7%) and that their child had been hospitalized for SCD complications before the age of 3 (*n* = 12, *M* = 80%).

Caregivers described other personal barriers that were not specifically asked about in the interview. The most discussed personal factors that interfered with their child's treatment were the caregivers' own medical conditions and the inability to miss work for appointments. One caregiver explained how their family manages medical appointments:

“Um, maybe my own like health issues and appointments, but I have been, so far everything has worked out as far as scheduling wise. Um, so if I have an appointment or an issue going on myself, then her dad would just step in. Um, it might cost him to take some time off work, but he would step in to make sure she gets her appointment.” (Caregiver 7)

This quote demonstrates a reality described by many caregivers – the problem solving required to prioritize their child's treatment. A caregiver explained how her efforts to balance her job with her child's medical treatment has led to negative internalizing:

“Having to lose so many jobs to take care of my child like it just makes you feel like nobody cares about your child but you. But you have to pay the bills so it is really depressing not being able to keep one.” (Caregiver 13)

Few characteristics of treatment were identified as problematic by caregivers who have obtained developmental services for their child (Table 2). All caregivers with experience with early intervention or another developmental service spoke positively about the treatment and providers. Positive comments included that early intervention was highly engaging to their child, that the providers were patient, that providers included them in activities, and that they observed improvement with their child.

Caregivers without experience with developmental services endorsed few concerns about the treatment itself or its accessibility, with two exceptions. Three caregivers expressed that they would expect the cost of the treatment to be a barrier to care, and two caregivers expected to feel misunderstood by a developmental provider. As one of them explained,

“I feel that providers don’t really take personal experiences into consideration when they’re offering treatments… like if you’re not just taking their advice or wanting to like give medicine that they’re suggesting for your child, I feel like they don’t really listen to your point of view.” (Caregiver 5)

#### 3.1.3 A positive caregiver-provider relationship facilitates access

Both providers and caregivers spoke about how the relationship between caregivers and providers facilitates caregiver access to services. Caregivers explained their desire to be heard and understood by providers, and providers explained how trust increases the likelihood that caregivers will pursue treatment for their child. Caregivers stated that providers need to listen to caregivers, make them feel comfortable asking questions, and ask families for feedback. One caregiver described the positive communication with her provider as follows:

“…they always make us feel comfortable… I guess, the way they communicate with us. Um, they tell us everything. They sit down. That’s the number one thing … They make us feel like, you know, we’re just having a, a regular conversation and it’s not uncomfortable” (Caregiver 2)

Providers also discussed the need for strong communication between caregivers and providers, and some emphasized the need for providers to speak clearly and concisely. One provider explained that, ideally, the relationship between providers and caregivers is one of partnership:

“I can learn from you and you can learn from me, kind of shared, um, thought partner model would help caregivers, um, kind of let their guard down and be open to, um, services and listening to what’s going on and really asking the parent or caregiver their experience and… reminding them, you know, they're the expert with their child and building up their confidence in the partnership and work.”(Provider 10)

Another provider explained how caregiver motivation, or “buy-in,” along with trust in providers, is essential for children who need early intervention services:

“I believe buy-in is essential when it comes to intervention support. One, the parent has to have buy-in that their child actually needs support. And then, two, that the parent has to have a trust factor that the provider is actually going to support their child in that area… I feel a way to alleviate that is for outreach to be paired in tandem with the referral, um, kind of like an appropriate handoff, you know, instead of a call this number.. kind of like, we're going to walk with you in this journey until everyone agrees that this is, this is happening.” (Provider 8)

As this provider noted, caregivers who are highly motivated to secure developmental services for their child may still struggle to navigate enrollment in early intervention or school services. Providers explained that developmental services are more accessible for patients who have a strong connection to a school or hospital system with staff dedicated to helping parents navigate those services. This statement was supported by caregiver anecdotes, as many caregivers who had obtained services for their child credited hospital staff or school educators for helping them schedule appointments.

### 3.2 Theme 2: caregiver knowledge and beliefs about SCD and development

#### 3.2.1 Breadth of knowledge about SCD varied by professional and personal experience

The most commonly discussed features of SCD were the associated pain and risk of stroke. Most providers could provide more precise answers about the developmental aspects than about the medical aspects of SCD, as most providers had a background in education rather than medicine. Providers identified several developmental delays that children with SCD may be at higher risk for, including issues with memory, attention, adaptive behavior, self-regulation, processing speed, social learning, speech/language, and motor skills. The concerns most often addressed by providers involved attention and speech. Providers also volunteered explanations for why such developmental delays may occur in children with SCD, including pain as a barrier to learning, time in the hospital or low attendance at school, and silent cerebral infarcts or stroke. Four providers were hesitant to attribute developmental delays to SCD itself, noting that the presence of delays in children with SCD is often confounded by other variables, such as low income or limited access to high-quality education. Regarding associated delays, one provider said,

“…a lot of it is, maybe not disease related, but complicated by disease.” (Provider 12)

Most caregivers (80%) had heard of hydroxyurea and more than half (53%) had heard of TCD. Most caregivers who were aware of hydroxyurea or TCD attributed their familiarity to their child's use of hydroxyurea or TCD. Caregivers also offered knowledge about hydroxyurea, with three noting that it can help with avoiding pain crises or hospitalization and three explaining that it is used to prevent blood cells from sickling. Caregivers were less descriptive about their understanding of TCD, with only two caregivers explaining its use to detect stroke.

Four caregivers were unaware that children with SCD are at a higher risk of delays in development than are their peers. Four other caregivers were aware that children with SCD are at risk for developmental delays but did not explain further. Three caregivers noted that speech may be impacted by SCD. Walking delays, difficulties with focus or memory, and problems with everyday activities were each mentioned once.

The mean accuracy score on the KIDI (total correct divided by total attempted) was 61% (SD = 0.07). In comparison, the mean KIDI accuracy score was 83% (SD = 0.16) among participants in a large study of infant/child development (*n* = 1,358), conducted in the same metropolitan area as the current study (38). Caregivers' accuracy on the KIDI was 1.78 SD below the accuracy observed in the prior study, suggesting knowledge gaps among our sample of SCD caregivers. The mean accuracy score of the current sample was also 1.20 SD below that of mothers whose children attended Head Start (*n* = 207, *M* = 70.7, SD = 11.4) (32).

#### 3.2.2 Knowledge comes from direct and professional experiences

Participants learned about SCD, developmental delays, or treatment for developmental delays in one of four ways: direct experience, professional experience, expertise of medical or educational professionals, or formal education. Direct experience was the most frequently discussed source of knowledge, with providers describing workplace interactions with children who have SCD and caregivers describing their own child or family member's experience. Most caregivers who were aware of TCD or hydroxyurea attributed this knowledge to their child's medical experience. Professional experience was largely discussed by providers, who learned about SCD by collaborating with other health professionals. Most caregivers cited expertise from medical or educational experts as their source of knowledge about SCD and development, with many emphasizing their reliance on the hospital's doctors and school program to learn about these topics. One caregiver described how her child's hospital shared information in a way that helped her to retain the new knowledge and build a relationship with hospital staff:

“Each visit that I go to [children’s research hospital], they teach me a little bit more every time. It’s kind of, it’s, it’s so much better than, you know, slamming all that knowledge on a parent when we first, you know, when we first get there.” (Caregiver 13)

Formal education as a source of knowledge about SCD was only mentioned by one provider and one caregiver.

The type and specificity of knowledge about SCD differed between providers and caregivers. Providers had a wider breadth of knowledge about potential risks of SCD, whereas caregivers had a more intimate knowledge of day-to-day life with SCD that was specific to their child's medical and developmental needs.

#### 3.2.3 Society lacks SCD awareness

Most providers did not endorse the statement that society lacks caring for those living with SCD. However, all providers who responded this way clarified their response, stating that people with SCD do not get the attention that they need from society. Nine of those providers said that there is a lack of knowledge about people living with SCD, and one provider described SCD as a low priority to those who disseminate knowledge:

"…most of the people with money and resources don't look like the people with sickle cell disease.. I can’t say it's a lack of caring about sickle cell disease, but more about… other priorities.” (Provider 12)

Most providers noted a lack of media coverage about SCD, and multiple providers linked the lack of knowledge and media coverage to the marginalization of Black people in society.

Most caregivers said there was not a lack of caring, but several noted that there is a lack of knowledge about the disease. Some caregivers further explained how the lack of knowledge in society is compounded by the invisible nature of the disease, such that others often trivialized their child's experience with the disease. One parent explained that her answer was informed by experiences of bias against her child with SCD at the hospital and at school. Caregivers almost unanimously said that there is not enough media coverage about SCD or representation of those living with the disease. One caregiver emphasized how the lack of awareness puts the burden on her to justify her child's needs:

“You mostly hear about children with cancer, you don’t too much hear anybody trying to help anybody with sickle cell… When I had my son, nobody, they, they heard of it, but they just don’t know anything about it. So, I’m having to constantly tell people what struggles he go through… It’s like nobody cares.” (Caregiver 13)

Providers and caregivers almost unanimously called for increased awareness of SCD and increased media representation for those who live with it.

### 3.3 Theme 3: caregiver preferences for developmental services

#### 3.3.1 Caregivers make decisions based on quality, provider trust, and accessibility

Providers and caregivers identified three factors that would influence caregiver acceptance and participation in a developmental education program for preschool-aged children with SCD. All 15 caregivers expressed interest in developmental education programs. Although interested, most caregivers cautioned that they would need to consider additional factors before committing to participate. When asked about how they would make a final decision about participation, their responses fell into the following categories: quality of the program, recommendations, and logistics. Caregiver responses regarding the quality of the program included discussions of the program's content and its perceived relevance to their child's needs. For example, one caregiver explained,

“…me personally, um, I just ask more about the program, what the program consists of… I would just want to see what I’m putting me and my son into it before I just jumped into the – I want to make sure it’s something good for him and not just okay for him.” (Caregiver 1)

Some caregivers placed high value on recommendations, explaining that they would be willing to try a program if it was suggested by a professional they trusted. Program accessibility was the most frequently discussed factor in decision making. Caregivers mentioned logistic considerations including work schedule conflicts, childcare needs, virtual or in-person participation, and time constraints. Providers endorsed the same decision-making considerations, but more frequently pointed out access issues such as lack of transportation or lack of technology.

#### 3.3.2 Logistic preferences vary by family

There was variability in how caregivers described the characteristics of a developmental education program that would be most accessible to their family, suggesting that no single format for intervention programming will meet all caregivers' needs. Although some caregivers were interested in virtual participation, other caregivers stated that they learn better in person. Similarly, some caregivers would prefer to meet regularly on a weeknight, but others stated that their work did not follow a traditional 9 am to 5 pm schedule. To accommodate the diverse needs of families, providers suggested offering virtual/in-person hybrid sessions, offering sessions at multiple times, and providing transportation or daycare to increase in-person attendance.

#### 3.3.3 Caregivers want SCD education and SCD community

Although caregivers had different preferences about the program logistics, they largely agreed on the desired qualitative aspects of the program. Caregivers frequently discussed development and school readiness from an SCD perspective, with a focus on elements to monitor in their child's development and risk factors common in children with SCD. They also discussed what to expect in kindergarten and how to teach their child to communicate their physical needs to teachers, particularly regarding staying hydrated throughout the day and communicating a pain crisis. One caregiver expressed anxiety about her child being in a larger class with less attention from the teacher:

“So, public school, you know, they don’t really baby them.. working with 20 plus kids… I know he’s not going to have as much attention on him, and I was paying attention to him… having a, a crisis since he’s so little he’s… still learning… what to tell me and how… he feels so that’s a concern of mine. But we’re working with him to try to communicate with us better…” (Caregiver 13)

Another recurring desire from caregivers was to learn about strategies to maintain their own mental health while coping with the stress of their child's transition to kindergarten. One caregiver explained this need:

“…I’d want more so, like, a support system to show, you know, manners in which that help as a, as a caregiver to, you know, keep moving forward and, you know, the steps that you can take then as much as you’re taking care of the children, but, like, giving us the education on how to keep ourselves whole in the midst of it.” (Caregiver 14)

Caregivers and providers discussed the idea of using a caregiver educational course as a support group. Multiple providers suggested using a coaching or cohort model rather than lectures. When asked who caregivers wanted to learn from, most suggested that the caregiver educational program be taught by a parent of a child with SCD. Caregivers expressed the need to hear from someone who has successfully gone through the process of preparing their child with SCD for school. Some caregivers also stated their willingness to hear from medical or educational professionals in the sessions. As one caregiver explained, parents should lead the session and then incorporate medical or educational professionals who can “back them up on the facts.” Regarding interventions for children, almost all participants agreed that the main criteria for instructors should be experience with early childhood education, but some caregivers suggested incorporating participation of older children with SCD as role models for the younger children.

## 4 Discussion

Patients with SCD comprise a historically marginalized population that face substantial health disparities (22, 39, 40). To establish an equitable developmental intervention for families of young children with SCD, we conducted a mixed-methods investigation through a health equity lens (25). Through analysis of both qualitative and quantitative data, we identified three broad themes that frame how caregivers and providers view developmental services (i.e., services to promote children's cognitive, language, and physical development). These themes include the quality of medical and educational experiences, caregiver knowledge and beliefs about SCD and development, and caregiver preferences for developmental services.

There were notable discrepancies in the barriers endorsed by caregivers and providers. Most caregivers did not endorse expecting or experiencing significant barriers to obtain developmental services for their child, whereas providers frequently endorsed numerous barriers for patient families. Providers described negative medical and educational experiences of caregivers. In contrast, caregivers mostly emphasized their own positive experiences. These discrepancies are consistent with prior literature assessing medical adherence in patients with SCD based on caregiver and provider reporting. Caregivers of children with SCD tend to overestimate rates of medical adherence (41, 42) and report fewer barriers to disease management (43) than do providers. Our results suggest that this response pattern extends to developmental services for children with SCD. Caregivers may not endorse these barriers due to stigma and fear of judgement or racism (44, 45). The differing lived experiences of providers and caregivers can lead to feelings of stigma and discrimination among families of patients with SCD (22). These feelings result in strained relationships and limited trust that can impact how barriers are reported (46). Furthermore, caregivers can only provide their own personal experience, whereas providers may have observed a wider range of barriers among caregivers. Conversely, although providers may have observed certain barriers in a few families, they may generalize these barriers to all families of children with SCD. Discrepancies in racial and ethnic backgrounds between providers and caregivers may also partially explain their experiences with these barriers.

Several caregivers highlighted the difficulties of balancing priorities when seeking developmental services for their child. For example, some caregivers noted that they often must prioritize their child's medical care or their own medical care, and others shared that it was difficult to maintain employment because their jobs do not provide the flexibility needed to attend appointments. Some caregivers (*n* = 3) and most providers (*n* = 12) shared that transportation or distance from the provider was a barrier to care. To overcome these barriers, developmental programs for children with SCD may prioritize virtual/telehealth services and offer more services outside working hours. Virtual/telehealth services may also have barriers due to limited internet access, but these issues can be addressed by providing hotspots or other low-cost internet options. Telehealth-based developmental services have been deemed feasible and acceptable for underserved and low-income families (47, 48). Telemedicine approaches for children and adolescents with SCD have received high levels of satisfaction from patients, providers, and caregivers (49–51).

Caregivers of young children with SCD showed limited knowledge of infant and child development, and providers identified these knowledge gaps as a barrier to accessing services. On a standardized questionnaire (KIDI) assessing parent knowledge of infant norms and milestones, principles of development, and parenting strategies, the caregivers in our study showed more limited knowledge than other caregivers living in the same metro area or caregivers with children enrolled in Head Start (32, 38). If caregivers are unfamiliar with normative expectations and principles of development, they may struggle to identify whether their child is delayed or to implement positive parenting strategies. Many caregivers were also unaware that children with SCD are at a higher risk of experiencing developmental delays or how these delays may manifest. Multiple providers reported observing these knowledge gaps and noted that a lack of knowledge about child development may limit follow-through with developmental services. Specifically, providers raised concerns that caregivers may not acknowledge that their child has a developmental delay or may not believe that such delays warrant further action. The discrepancy between caregivers' perceptions and normative expectations highlights a significant barrier to the utilization of developmental services. If caregivers are referred for a developmental evaluation or service, follow-through may be poor if they do not view their child's development as a concern. Caregivers of young children with SCD need education on normative child development and parenting strategies, but knowledge alone is unlikely to lead to significant behavior change (52). Instead, providers can use strategies such as motivational interviewing (53, 54) to promote caregivers' self-efficacy (55, 56) allowing caregivers to feel confident in their ability to follow through with provider recommendations. Culturally tailored parent education programs that utilize motivational interviewing and emphasize cultural pride may be particularly effective (57, 58).

Although providers and caregivers gave contradictory responses about barriers to developmental services, the two groups agreed on facilitators to participation. Both groups highlighted the importance of a positive caregiver–provider relationship to facilitate access to developmental services. Previous studies have documented distrust and poor communication between families of patients with SCD and their providers (59–61). Poor caregiver–provider communication is associated with more hospitalizations and worse health literacy (62). Thus, to facilitate access and engagement in developmental services, the medical team and developmental providers must use communication strategies that build trust. This may be accomplished by implementing family-centered communication, which involves obtaining and understanding the families' perspectives, considering psychosocial and cultural context, and reaching a shared understanding of health concerns and treatment options (59, 63). Interventions emphasizing family centered communication engage families as partners in designing and implementing interventions and seek routine feedback.

Few caregivers reported symptoms of depression on the PROMIS and most denied stress or depression as a barrier to accessing services. In contrast, most providers endorsed caregiver stress and/or depression as a barrier to accessing services. Compared to normative expectations, both caregivers of (64) and child/adolescent (65) patients with SCD do not display elevated rates of depression. Yet, caregiver stress and depression is associated with their child's pain intensity and functional impairment (64), which may become more apparent as their child grows older. Assessing caregiver mental health and promoting coping strategies should be incorporated into family-centered communication (59, 63) with providers.

Caregivers consistently endorsed a strong preference to learn from other families and to build a community of caregivers with shared experiences. The desire to learn from other caregivers or patients with SCD is consistent with findings from other studies (66, 67). These findings may reflect cultural values, as African Americans families tend to be more communalistic and value interdependence more than do European American families (68). Caregivers noted that other families with SCD can comment on the challenges of the lived experience and provide solutions that are practical and approachable. Caregivers expressed a desire to interact with other families for social connectedness and community building, as they experience feelings of isolation related to their child's SCD diagnosis and have limited interactions with other families with shared experiences. These feelings of isolation were exacerbated by the perception that there is a lack of caring or media coverage of SCD within the hospital system and broader society, due in part to racism. Caregivers expressed the burden of having to frequently educate others about SCD. Families living with a chronic disease may develop a strong social identity related to the disease (69–71). Peer-to-peer or group interactions that build upon this identity provide a sense of shared meaning, support, and efficacy that can reduce depression and loneliness (72). Thus, developmental services that foster these connections among caregivers may have better engagement and may more successfully promote caregiver self-efficacy and mental health.

By collecting information from both providers and caregivers, we obtained unique perspectives on developmental services for young children with SCD. The convergent mixed-methods approach incorporating standardized quantitative assessments and qualitative data comprehensively captured potential barriers and facilitators and caregiver knowledge of developmental norms. However, several study limitations exist. Not all caregivers had experience with developmental services and therefore could only relay expectations based on medical care or preschool experiences for their child. We intentionally included caregivers with or without these exposures to gain perspectives from a diverse sample of caregivers rather than from only those who had concerns about their child's development. All data were collected from caregivers served at a single institution and from providers in the surrounding area, potentially limiting generalizability of the results. Caregiver and provider experiences may differ based on geographical location, hospital resources, and broader community resources. Therefore, further investigation is needed through multi-center studies using diverse geographic sampling to evaluate if results are consistent across centers.

## 5 Conclusions

Patients with SCD and their families experience health disparities that negatively impact quality of life. Using the Health Equity Implementation Framework (25), we identified determinants of access to and quality of developmental services according to caregivers and providers. There were notable discrepancies in barriers to care based on caregiver and provider responses. Caregivers of young children with SCD showed limited knowledge of infant and child development, and providers identified these knowledge gaps as a barrier to accessing and utilizing services. To increase engagement and follow-through of developmental services, interventions should use family-centered communication to facilitate shared understanding and incorporate peer support to foster self-efficacy among caregivers. These findings will support the creation of accessible developmental interventions for children with SCD.

## Data Availability

The raw data supporting the conclusions of this article will be made available by the authors, without undue reservation.

## Supplementary material

Supplementary File 1 Caregiver interview guide. This semi structured interview guide was used with each of the caregivers interviewed for the present study.

Supplementary File 2 Provider interview guide. This semi structured interview guide was used with each of the providers interviewed for the present study.
