# Supplementary material for: Caregiver and provider perspectives on developmental services for children with sickle cell disease: a mixed methods analysis
Source: Front Pediatr. 2025 Mar 21;13:1530457. doi: 10.3389/fped.2025.1530457 (PMC11968433; doi:10.3389/fped.2025.1530457)
Supplement: Supplementary File 1 — Caregiver interview guide. This semi structured interview guide was used with each of the caregivers interviewed for the present study. [file Datasheet1.docx]

**Interview guide: Caregivers**

Thank you for speaking with me today about your experiences with your child’s care related to SCD. We appreciate your time and we look forward to your input. The purpose of this interview is to help plan an intervention to address developmental or learning difficulties in preschool children with sickle cell disease. Based on your feedback, we hope to start a school readiness program next summer. Your opinions and experiences will help us design our program.

There are no right or wrong answers— We are interested in your experiences and opinions. We will be asking questions about your experiences related to your child’s development and interest in intervention services.

The interview will be recorded and should take about 45 minutes. If you have any questions throughout or after the interview, please let me know. We can stop the interview at any point, if necessary. Do you have any questions before we begin?

**1. KNOWLEDGE OF SICKLE CELL DISEASE NEURODEVELOPMENTAL RISK**

What do you know about developmental or learning difficulties children with sickle cell disease might experience?

[*Prompt if needed*] Developmental or learning difficulties can include delayed language or motor milestones or problems with thinking, attention, and learning that can affect academic readiness, schoolwork, and behavior. Children with sickle cell disease are at risk for these problems.

1. How did you first learn about developmental or learning difficulties experienced by some children with sickle cell disease?

Medical Visit Independent Research During this discussion Other

1. Has your child experienced developmental delays or any problems in learning, language, attention, or thinking skills?

Yes No

Please describe. [*If yes, try to get information on when problems were first noted and areas of concern: language, motor, thinking, behavior, academic skills with examples*.]

1. Have you heard of a drug called hydroxyurea?

Yes No

[*If yes*] Please tell us what you know about hydroxyurea

[*If no*] - Hydroxyurea makes red blood cells bigger. It helps them stay rounder and more flexible and makes them less likely to turn into a sickle shape. The medicine does this by increasing a special kind of hemoglobin called hemoglobin F, also called fetal hemoglobin because newborn babies have it.

[*if unaware of the relationship between Hydroxyurea and the brain*] In patients with sickle cell disease, hydroxyurea can also protect the brain from insults such as stroke and limit the developmental and learning difficulties that patients with sickle cell disease experience.

Is this your first time hearing about the relationship between hydroxyurea and the brain?

Yes No

Does this information have any impact on your decision to have your child receive hydroxyurea?

1. Have you heard of an assessment called a transcranial doppler screening?

Yes No

[*If yes*] Please tell us what you know about transcranial doppler screening.

[*If no*] Transcranial Doppler screening is used to detect children with sickle cell anemia who are at risk for stroke. It is able to detect risk of stroke by using ultrasound to measure the velocity or speed of blood flow through the blood vessels in the brain.

**2. EARLY LEARNING ENVIRONMENT AND INTERVENTION**

How have you prepared your child to enter kindergarten?

1. Has your child ever attended day care?

Yes No

# Has your child ever attended or are there plans to attend:

Head-start Preschool Neither

[If neither, assess reasons for not attending]

Have you ever reached out to an early childhood professional in medicine or education to learn more about getting services to help your child with learning or developmental difficulties?

# Now I’m going to ask about some specific types of services and whether your child with sickle cell disease has ever received them. Has your child received-

[*ask about each developmental service listed below*]*.*

| Physical Therapy | Yes | No |
| --- | --- | --- |
| (Physical therapists teach exercises or guided movements to improve a child’s strength, coordination, or balance. Treatment usually occurs at least weekly and can take place at home or in a childcare or healthcare environment). |  |  |
| Occupational Therapy | Yes | No |
| (Similar to physical therapy but focuses on the child’s ability to perform tasks at home and school. Therapists may work on motor skills, hand-eye-coordination, or other skills so that children can meet goals like holding utensils, buttoning their clothes, etc. This type of service can take place at home or in a childcare or healthcare environment) |  |  |
| Speech Therapy | Yes | No |
| (Speech therapists use exercises and activities with the child to treat delays in processing language or producing language vocally. This type of service can take place at home or in a childcare or healthcare environment) |  |  |
| Developmental Therapy | Yes | No |
| (Therapy for children three-years-old or younger with developmental delays or disabilities. Therapists work with children and parents to help children meet developmental milestones. Often therapy services are offered at home or elsewhere in the community) |  |  |
| Behavioral counseling or therapy | Yes | No |
| (Could be provided by psychologists or social workers. Therapists may use a variety of techniques with children and their caregivers to treat behavioral disorders. This type of service can take place at home or in a childcare or healthcare environment) |  |  |
| Psychological testing | Yes | No |
| (Interviews and tests administered in a clinic to determine a child’s strengths and weaknesses. May assess intelligence, achievement, memory, attention, behavior, emotional and social development.) |  |  |
| Medication | Yes | No |
| Medications to manage their behavior, help them focus, or aid their development  Other: |  |  |

**3. BARRIERS/FACILITATORS**

Thank you for sharing all that information about your knowledge and experience. Next, we would like to share some lists of things that other caregivers have said makes it harder to obtain therapy for developmental or learning difficulties for their child with sickle cell disease.

Do you have any questions about what these therapies look like before we start? [*clarify based on section 2.C*].

|  | [*If the caregiver said yes to receiving any type of service from 2.C or that they have tried to obtain services*] | [*If the caregiver previously stated that they’ve never tried to get their child services*] |
| --- | --- | --- |
| **Introduction** | Thinking about when your child has received [*therapy identified in section 2C*], you will say yes if you have experienced any of these issues and no if you haven’t.  OR  *If they endorsed attempting to obtain services:*  Thinking about when you tried to get (specific service) for your child, please tell me yes if you received any of the following issues and no if you didn’t. | So you’ve never looked into obtaining any services for your child like physical therapy, speech, or any other type of developmental service, is that right?  For this next part of the interview, please imagine that your child required the services we just discussed (e.g., physical, speech, developmental therapy), and tell us whether you would expect any of the following to happen |
| **Innovation**  ***The treatment itself***  ***Other***  ***Innovation Factors***  ***Clinical Encounters*** | 1. When your child received therapy services, did it take too much time? 2. Did you feel like you understood the therapy and what it was for? 3. Did you believe the therapy was needed? 4. Were there concerns about the cost of the therapy?   *If any barriers endorsed:* How might you overcome the (barrier)? Is there anyone or anything that makes (barrier) easier? | So based on your experience with your child’s sickle cell treatment more generally,   1. would you expect developmental therapy to take too much time? 2. Do you feel like you would know enough about the therapy? 3. Do you think therapy is necessary? 4. Would you be concerned about the cost of therapy services?   *If any barriers endorsed:* How might you overcome the (barrier)? Is there anyone or anything that makes (barrier) easier? |
|  | 1. Was it hard to learn about the therapy initially? 2. Was it hard to get an appointment? 3. Were there long wait times to get an appointment? 4. Anything else about the therapy?   *If any barriers endorsed:* How might you overcome the (barrier)? Is there anyone or anything that makes (barrier) easier? | 1. If your child was in need of one of these services, do you think someone would tell you about them? Who would you expect to tell you? 2. Would you expect there to be long wait times to make an appointment?     *If any barriers endorsed:* How might you overcome the (barrier)? Is there anyone or anything that makes (barrier) easier? |
|  | Now let’s think about clinical encounters. Thinking about any time a professional in the clinic talked to you about your child’s development or learning,   1. Did you ever have problems when you met with a provider? 2. Did you ever feel that you were not being understood by your provider? 3. How could providers make families feel more comfortable during a clinical encounter? 4. Is there anything else you want to share about meeting with providers? | |
| **Recipients**  ***Patient Factors***  ***Provider Factors*** | Next I’m going to ask you about your clinical experiences. Tell me if you think any of these things have ever been a barrier to getting your child treatment for a learning or developmental delay:   1. Your motivation to get treatment for your child 2. Stress or depression in your life 3. Housing or your living situation 4. Your cultural group’s views on learning difficulties or developmental delays 5. Trusting your providers 6. Negative things you heard about treatment from other people 7. Distance from hospital/clinic 8. Transportation   Is there anything else in your personal life that might make it harder for you to get learning or developmental services for your child? | |
|  | This third list includes things about your developmental providers (therapists, psychologists, school personnel).  Did you feel like your provider was   1. too busy to provide quality therapy 2. wasn’t answering your questions   Did a provider ever say your child should not get therapy?  Anything else about your providers?  Have you had good experiences with providers helping you through your child’s therapy for developmental or learning delays? What did that look like? | Based on your experience with other clinicians, would you expect developmental providers (therapists, psychologists, school personnel) to-   1. be too busy to provide quality therapy 2. not answer your questions   Anything else you’d expect about a developmental provider? |
| **Context**  ***Inner Context*** | Still thinking about when you interacted with developmental providers-   1. Have you ever had a bad experience trying to obtain services? 2. Did you ever notice that there were no providers available in your community? 3. Did you ever notice that it’s harder to get treatment in your community?   Anything else about your community?  How could the local community help address developmental or learning difficulties experienced by patients with sickle cell disease? | Again, based on your experience with other clinicians-   1. Would you expect to have a bad experience trying to obtain services? 2. Would you expect to have difficulty finding providers available in your community? 3. Have you ever noticed that it’s harder to get treatment (in general) in your community? |
| ***Organizational Level*** | Now I’m going to ask you specifically about [children’s hospital] and developmental services. Do you feel that [children’s hospital]-   1. doesn’t seem like they want to help patients with developmental problems? 2. has not offered such services?   Anything else about [children’s hospital]?  How could [children’s hospital] address developmental or learning difficulties experienced by patients with sickle cell disease? | |
| ***Outer Context*** | Do you think in society in general that there’s a-     1. Lack of caring about patients living with sickle cell disease? 2. No media coverage on about sickle cell disease treatment?   Anything else about society or environment, at large?  Do you feel like information you see about sickle cell disease or treatment for sickle cell disease applies to your child?  How could society help address developmental or learning difficulties experienced by patients with sickle cell disease? | |

# Open Ended Health Disparity Barriers Question

*If applicable*: On the questionnaire you stated you were (insert Black or African American). How does that relate to getting speech, physical, occupational or developmental therapy that could help with developmental or learning difficulties for your child? Did you ever feel you were treated differently trying to get these therapies?

**4. IDEAS ABOUT TREATMENT**

My final set of questions is about how you think [children’s hospital] could improve programs for developmental or learning difficulties for children with sickle cell disease

- Besides things you’ve already shared with me, specifically, what would you want to see [children’s hospital] or providers at [children’s hospital] do to better help with developmental problems in children with Sickle cell disease?
- If [children’s hospital] were to provide a new program for these problems, how would you decide if you want to participate?

Now I will describe potential interventions to improve children’s early learning skills in preparation for kindergarten.

**Parent Training** (*please be sure to convey all points when describing the intervention)*

- Parents are taught strategies (e.g., teaching through encouragement) to help children with learning and behavior, and given resources to help development and early learning [one or both parents]
- Parents attend workshops weekly in the summer
- Can be completed virtually or in-person

1. Would you be interested in participating?

Yes  No – Why?

time  do not require coaching  other (specify)

1. Would you rather participate virtually (at-home) or in-person?

Virtually  In-person

[Prompt for reasoning]

C. If we created a Parent Training Program….?

[*Prompt for the information below*]

- When would it occur? Prompt with suggestions if “I don’t know” for each question
- Where would it occur?
- For how long?
- What content to focus on?
- Who would you like to learn from?

**Summer Classroom-Based Intervention** (*please be sure to convey all points when describing the intervention)*

- Classroom teachers work with kids to work on and improve development of early learning skills and school readiness at [children’s hospital]
- Children attend 3 sessions a week for 8 weeks before the start of kindergarten (during the summer)

A. Would you be interested in your child attending such a classroom?

Yes  No – Why?

time  transportation  other (specify)

B. If we created a Summer Classroom-Based Intervention…?

[*Prompt for the information below*]

- When would it occur (during the summer)?
- Where would it occur?
- For how long?
- What content should we focus on?
- Who would lead the intervention?

Is there anything else you’d like to add?

Do you have any concerns or questions for me at this time?
