# Supplementary material for: Caregiver and provider perspectives on developmental services for children with sickle cell disease: a mixed methods analysis
Source: Front Pediatr. 2025 Mar 21;13:1530457. doi: 10.3389/fped.2025.1530457 (PMC11968433; doi:10.3389/fped.2025.1530457)
Supplement: Supplementary File 2 — Provider interview guide. This semi structured interview guide was used with each of the providers interviewed for the present study. [file Datasheet2.docx]

**Interview guide: Providers**

Thanks for participating in this interview. We appreciate your time, and we look forward to your input. The purpose of this interview is to help plan an intervention to address developmental or learning difficulties in preschool children with sickle cell disease. There are no right or wrong answers—it is your observations and opinions that we are interested in.

The interview will be recorded and should take about 45 minutes. If you have any questions throughout or after the interview, please let me know. We can stop the interview at any point, if necessary. Do you have any questions before we begin?

**Self-intro & familiarity with SCD**

- Tell me about your role with [organization name]
- To give us some general background about your work, could you briefly talk about your experiences working with patients with SCD

**1. KNOWLEDGE OF SICKLE CELL DISEASE NEURODEVELOPMENTAL RISK**

- What is your understanding of developmental or learning difficulties children with sickle cell disease might experience?

[*Prompt if needed*: Developmental or learning difficulties can include delayed language or motor milestones or problems with thinking, attention, and learning that can affect academic readiness, schoolwork, and behavior. Children with sickle cell disease are at risk for these problems.]

- What developmental or learning difficulties, if any, have you observed in children with SCD?
- From your experience, how are developmental or learning difficulties typically addressed for patients with SCD? [Try to steer towards interventions/services rather than assessment]

**2. BARRIERS/FACILITATORS**

Thank you for sharing all that information about your knowledge and experiences with sickle cell disease. Next, we would like to share some lists of things that potentially make it harder to obtain professional help for developmental or learning difficulties for children with sickle cell disease. Please say yes if you have observed any of these troubles and no if you haven’t. Are you ready?

| **LEVEL** | **LIST OF BARRIERS**  **(ask each one)** |
| --- | --- |
| **Innovation**      ***The treatment itself***                ***Other innovative factors***                ***Clinical Encounter*** | This first list is about caregivers’ experiences with developmental services    Thinking specifically about developmental services, have you observed or have caregivers told you that-   1. the treatment took too much time 2. caregivers did not understand the treatment 3. caregivers did not believe treatment was needed 4. the cost of treatment was prohibitive     How might the family overcome the (barrier)? Is there anyone or anything that makes (barrier) easier? [*Ask for all identified barriers]*    Do you know of caregivers-   1. being unaware that the treatment existed 2. not believing the treatment would work 3. struggling with wait times to get an appointment 4. struggling with previous negative experiences with treatment 5. anything else about treatment for developmental or learning difficulties.     I have some more questions now about clinical encounters.  Have you observed-   1. problems when the patient met with a provider 2. the caregiver felt misunderstood by the provider 3. anything else about meetings with providers?     How could providers make families feel more comfortable during a clinical encounter? |
| **Recipients**  ***Caregiver preferences***  ***Provider preferences*** | This next list involves things about the patients’ caregivers and their experiences.    Have you seen any of the following caregiver preferences impact treatment or services?   1. Their motivation to get treatment or services 2. Experiences of stress or depression 3. Their housing or living situation 4. How their cultural group views learning difficulties or developmental delays 5. Trusting providers 6. Experiences stigma about their child having developmental or learning difficulties 7. Negative things they heard about treatment or services from other people 8. Distance from hospital/clinic 9. Transportation 10. Anything else about these families?     This next list includes things about providers (therapists, psychologists, school personnel)    Have you ever observed-   1. Providers to be too busy 2. Providers didn’t answer the patient’s questions 3. Anything else about your providers? |
| **Context**  ***Inner***  ***Society*** | This next list includes things about the community    Do you think that within the caregiver’s community-   1. Caregivers had bad experiences trying to obtain services 2. Limited providers available in the community 3. Anything else about the community?     This last list of things is about the greater environment and society.    Do you think that in society in general there is a-   1. Lack of caring about patients living with sickle cell disease 2. No media coverage about sickle cell disease treatment 3. Anything else about society or environment, at large?   How could the local community help address developmental or learning difficulties experienced by patients with sickle cell disease? |

# Open Ended Health Disparity Barriers Question

The majority of patients treated for sickle cell disease are Black/African American. How does that relate to getting treatments for developmental or learning difficulties for these children? Have you ever felt that these patients were treated differently?

**3. IDEAS ABOUT TREATMENT**

My final set of questions is about how you think [children’s hospital] could improve treatments for developmental or learning difficulties for children with sickle cell disease.

- - Besides things you’ve already shared with me, specifically, what would you want to see [children’s hospital] or providers at [children’s hospital] do to better treat developmental problems in children with sickle cell disease?
  - If [children’s hospital] were to provide a new treatment for these problems, how do you think families would decide if they wanted to participate?

Now I will describe potential interventions to improve early learning skills.

**Parent Training** (*please be sure to convey all points when describing the intervention)*

- Parents are taught strategies to help children with learning and behavior, and given resources to help development and early learning [one or both parents]
- Parents attend workshops weekly in the summer
- Can be completed virtually or in-person

A. Do you think families would be interested in participating?

Yes  No – Why?

time  coaching is not needed  other (specify)

B. Would families rather participate virtually (at-home) or in-person?

Virtually  In-person

[Prompt for reasoning]

C. What would an ideal Parent Training Program look like to you?

[*Prompt for the information below*]

- When would it occur?
- Where would it occur?
- For how long?
- What content to focus on?
- Who would parents like to learn from

**Summer Classroom-Based Intervention** (*please be sure to convey all points when describing the intervention)*

- Classroom teachers provide instruction known to improve development of early learning skills and school readiness at [children’s hospital]
- Children attend 3 sessions a week for 8 weeks before the start of kindergarten (during the summer)

A. Do you think families would be interested in their child attending such a classroom?

Yes  No – Why?

time  transportation  other (specify)

B. What would an ideal Summer Classroom-Based Intervention look like to you?

[*Prompt for the information below*]

- When would it occur (during the summer)?
- Where would it occur?
- For how long?
- What content to focus on?
- Who would lead the intervention?

Is there anything else you’d like to add?

Do you have any concerns or questions for me at this time?
